# Direct comparison of different surgical approaches in a woman with bilateral osteochondrosis dissecans of her knees: a case report

**DOI:** 10.1186/s13256-015-0796-0

**Published:** 2016-01-19

**Authors:** Marco M. Schneider, Stefan Preiss, Gian M. Salzmann

**Affiliations:** Schulthess Clinic, Musculoskeletal Centre, Lengghalde 2, CH-8008 Zurich, Switzerland

**Keywords:** Knee, OCD, OD, Osteochondrosis dissecans, Reconstruction, Refixation, Single patient

## Abstract

**Background:**

Osteochondrosis dissecans is a disorder of the subchondral bone potentially affecting the adjacent articular cartilage. There remains disunity with regard to treatment methods.

**Case presentation:**

We present the case of a 21-year-old Swiss woman who presented with clinically symptomatic bilateral osteochondrosis dissecans lesions at both medial femoral condyles. She underwent sequential surgical intervention and was prospectively monitored using clinical scores and magnetic resonance imaging. Her left knee was treated with an open refixation of the osteochondrosis dissecans lesion with two 2.0 mm screws in combination with a cancellous bone graft and subchondral drilling since the cartilage of the osteochondrosis dissecans lesion was intact. On her right knee, she underwent open removal of the defective bone and cartilage, cancellous bone graft with subchondral drilling and coverage with a bilayered collagenous membrane (autologous matrix-induced chondrogenesis technique) since the cartilage of the osteochondrosis dissecans lesion was not intact. At final follow-up 12 months after surgery her Lysholm score had improved from 79 to 95 on her left side and from 74 to 78 on her right. Magnetic resonance imaging displayed good integration of the cancellous bone graft with a slight irregularity at the articular surface on her left side (magnetic resonance observation of cartilage repair tissue (MOCART) 75). The magnetic resonance imaging of her right knee depicted satisfying bony reconstitution with yet more irregularity at the joint surface (magnetic resonance observation of cartilage repair tissue 65) in comparison to her left femoral condyle.

**Conclusions:**

In cases of failed conservative treatment of osteochondrosis dissecans lesions of the knee joint surgery should be taken into consideration. Considering this case, we believe that the focus should be the preservation of the cartilaginous layer whenever possible or at least replacement with high quality replacement tissue, such as autologous chondrocyte implantation.

## Background

Osteochondrosis dissecans (OD) is a disorder of the subchondral bone potentially affecting the adjacent articular cartilage that may lead to the detachment of cartilage and bone fragments. The disease is classified into two forms: a juvenile and an adult form [1, 2]. Several etiologies for OD have been described: direct trauma including repetitive microtrauma, genetics, inflammation, vitamin imbalance and vascular abnormalities [1, 3–6]. A study by the European Pediatric Orthopedic Society investigated the epidemiology of OD: the incidence for boys was two times higher than for girls, in 12.6 % cases bilateral OD was detected and no difference in frequency between left and right side could be found [7]. The lateral aspect of the medial femoral condyle is thought to be the main location across the knee joint with repetitive microtrauma being the main etiology [8–10]. OD can be divided into different stages by various classifications [7, 11–15]. One of the classifications was described by Bruns, in which lesions are graduated in four stages using plain radiographs, magnetic resonance imaging (MRI) or arthroscopy [16]. We used the MRI version since it displays the most frequently used imaging modality in the diagnostics of OD: I = beginning of osteolysis and bone marrow edema; II = sclerosis and osteolysis/osteonecrosis; III = loose body without dislocation, fluid collection, disruption of cartilage; IV = empty OD site, dislocated cartilage body, effusion. The outcome of OD of the knee joint is mainly dependent on the duration and stage (and therefore the stability) of the lesion [17]. Juvenile OD is associated with less instability and therefore a higher response to conservative treatment [10]. Furthermore, it has been reported that children and adolescents have greater healing potential in comparison to adult patients. Surgical management in patients with failed conservative treatment can be important since OD is related to a high risk of osteoarthritis [18, 19]. At present, no golden standard for optimal operative OD management has been accepted. However, a variety of procedures are on offer to the surgeon [17]. We present the case of a patient who presented with clinically symptomatic bilateral medial-sided knee joint OD and underwent two distinct surgical procedures.

## Case presentation

**Fig. 1:**
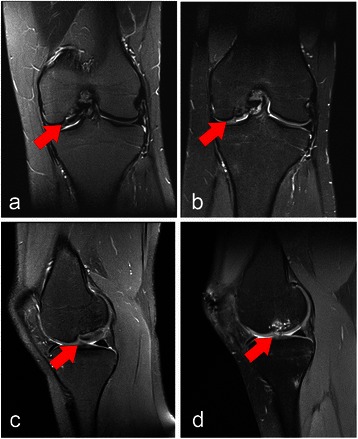
Preoperative and postoperative magnetic resonance imaging of the patient’s left knee. Coronal (**a** and **b**) and sagittal (**c** and **d**) t2-weighted magnetic resonance imaging images of her left knee preoperative with osteochondral lesion (see arrows) and 12-month postoperative with refixation of the lesion showing the repaired tissue (see arrows)

A 21-year-old Swiss woman with a radiological-controlled bilateral straight leg axis presented at our department after 10 years of non-operative treatment of an OD lesion at the medial femoral condyle of her left knee. Her main complaints were an intermittent blocking of her knee joint and discomfort during and after arduous activities. A MRI revealed an OD lesion grade III according to Bruns [16]. The cartilage surface at the OD site was intact on MR images (see Fig. 1). After thoughtful case evaluation and discussion with the patient we considered that conservative management was no longer effective and indicated surgery.

The operative therapy consisted of an initial arthroscopy. The cartilage layer at the OD location was intact. Yet, under probe testing it was unstable and could be partially detached from the subchondral bone. We did not consider retrograde drilling which can be indicated in grade III lesions. We then continued with a mini-open arthrotomy of her left knee and detachment of the OD lesion (1 × 2 cm), which presented with healthy appearing cartilage but necrotic bone, leaving a medial hinge. This was followed by debridement of the subchondral bone until all necrotic bone was removed using a motorized burr, deep drilling (1.2 mm drill) into the subchondral bone/medial condyle (“vitality drilling”), implantation of a cancellous bone graft, relocation of the cartilaginous OD fragment and refixation with two 2.0 mm (24 mm in length) titan screws. The quality of the surrounding cartilage was good and without lesions. The border to the origin of the posterior cruciate ligament was intact with good containment. The OD fragment was fixed in a stable manner. She was free of pain 6 months postoperatively. Following a MRI and X-ray analysis of her left knee joint we performed arthroscopic removal of the two screws. The OD fragment appeared well integrated without signs of instability and the cartilage seemed healthy under probing. No damage at the opposing tibia, meniscus or surrounding cartilage was found. Immediately before and 2 weeks after the implant removal she was able to perform sporting activities such as biking and jogging.

**Fig. 2:**
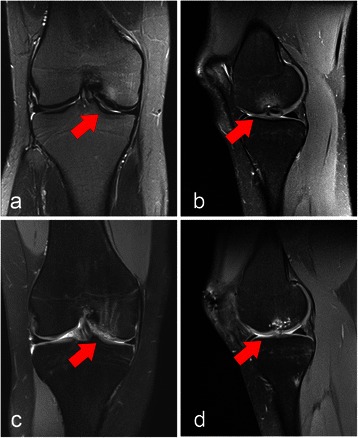
Preoperative and postoperative magnetic resonance imaging of the patient’s right knee. Coronal (**a** and **c**) and sagittal (**b** and **d**) t2-weighted magnetic resonance imaging images of her right knee preoperative with osteochondral lesion (see arrows) and 12-month postoperative with repair tissue (see arrow)

During rehabilitation a MRI of her right knee was performed because of rising complaints during activities. The examination displayed another OD lesion (grade III according to Bruns) of the medial femoral condyle of her right knee joint (see Fig. 2). Symptoms at her right knee joint increased constantly during rehabilitation of her left knee joint. We initiated conservative management of her right knee joint, which improved her symptoms, but she had to significantly reduce sporting activity due to pain and locking. A full return to sport was impossible because of her right knee joint symptoms. Since complaints and morphology of the OD lesion were similar to the contralateral side, the same type of operation was planned. During arthroscopy the cartilage of the OD lesion appeared damaged with a rough surface (Outerbridge grade II to III) when compared to her contralateral knee joint. Probe testing revealed instability of the lesion with, and different to her left knee joint, fissures and cracks within the cartilage. Arthrotomy was commenced. During inspection the OD lesion (2 × 1 cm) cartilage presented with advanced damage of the cartilage layer and necrotic bone. Retrieval of the OD fragment was impossible. Therefore, the OD fragment was discarded and we decided to use a modified autogenous matrix-induced chondrogenesis (AMIC). The necrotic bone was removed; deep drilling (1.2 mm K-wire) into the subchondral bone/medial condyle was performed to provoke bleeding. Implantation and impaction of a cancellous bone graft was applied and the construct was covered with a bilayer type 1/3 collagenous membrane (ChondroGide, Geistlich, Wollhusen, Switzerland), which was sutured to the surrounding cartilage using 6.0 interrupted sutures and sealed by the use of fibrin glue. The surrounding cartilage was intact. The border to the origin of the posterior cruciate ligament was intact with good containment.

Rehabilitation protocols differed. For her left knee full weight bearing was allowed 1 week after the operation, whereas partial weight bearing was recommended for 6 weeks after surgery on her right knee. Return to sports with full contact sports was allowed 4-6 months after refixation and 12 months after reconstruction with the AMIC technique. The rehabilitation did not proceed as fast as it did with her left knee. During the 12 months follow-up she still complained about recurrent instability during daily activities as well as temporary pain. She reported that both rehabilitation and pain reduction were significantly delayed in comparison to her contralateral knee joint. The 12-month Lysholm score for her left knee (95) documented a higher satisfaction in the follow-up examination in comparison to her right knee (78; see Table 1). Her magnetic resonance observation of cartilage repair tissue (MOCART) score was used to evaluate the articular cartilage repair tissue 12 months postoperatively (see Table 2). The MRIs of both knees preoperative and postoperative are shown in Figs. 1 and 2.

**Table 1:** Lysholm score for both knees preoperatively, 6 and 12 months postoperative

	Left knee			Right knee		
	Pre	6-mo FU	12-mo FU	Pre	6-mo FU	12-mo FU
Limping	3	3	5	3	1	3
Weight bearing	5	5	5	5	5	5
Blocking	6	15	15	15	10	15
Instability	25	25	25	15	10	10
Pain	15	20	20	15	0	20
Swelling	10	10	10	6	6	10
Climbing stairs	10	10	10	10	10	10
Crouching	5	5	5	5	5	5
Total	79	93	95	74	47	78

*FU* follow-up, *mo* month, *pre* preoperative

**Table 2:** Magnetic resonance observation of cartilage repair tissue assessment: morphological magnetic resonance imaging grading and point scale of both knees 12 months postoperative

Variables	Left knee	Right knee
1. Degree of defect repair and filling of the defect	20	15
Complete (20)		
Hypertrophy (15)		
Incomplete		
>50 % of the adjacent cartilage (10)		
<50 % of the adjacent cartilage (5)		
Subchondral bone exposed		
2. Integration of the border zone	10	15
Complete (15)		
Incomplete		
Demarcating border visible (slit like; 10)		
Defect visible <50 % of the length (5)		
Defect visible >50 % of the length (0)		
3. Surface of the repair tissue	5	5
Surface intact (10)		
Surface damaged <50 % of depth (5)		
Surface damaged >50 % of depth (0)		
4. Structure of the repair tissue	0	0
Homogeneous (5)		
Inhomogeneous (0)		
5. Signal intensity of the repair tissue	30	15
Normal (identical to adjacent cartilage; 30)		
Nearly normal (slight areas of signal alteration; 15)		
Abnormal (large areas of signal alteration; 0)		
6. Subchondral lamina	0	0
Intact (5)		
Not intact (0)		
7. Subchondral bone	0	5
Intact (5)		
Not intact (0)		
8. Adhesions	5	5
No (5)		
Yes (0)		
9. Effusion	5	5
No (5)		
Yes (0)		
TOTAL	75	65

## Discussion

We present a case of bilateral osteochondral lesions of the medial femoral condyle in a young and active patient. Since the incidence of bilateral OD lesions is reported to be up to 29 % a radiographic evaluation of the contralateral side is recommended by selected authors [20]. Yet, examinations among asymptomatic patients always require thoughtful considerations.

Plain radiographs and MRI of the knee joint remain the diagnostics of choice [21]. Marlovits *et al*. developed a cartilage repair tissue grading scale (MOCART), which helps to evaluate the reintegration of the cartilage after operative treatment [22]. In addition, leg alignment should be taken into consideration. Usually, a conservative treatment leads to satisfactory results [21, 23–25]. In our case we performed surgery on both knees after failed conservative treatment. For our patient’s left knee we were able to reattach the OD lesion with two screws, whereas the cartilage on her right side was damaged so that only the use of a cancellous bone graft in combination with a collagenous membrane was possible.

Despite our operative approaches, surgery can be performed in various ways when conservative treatment fails or the lesion appears to be unstable or detached. Each treatment has its limitations and as of today no surgical approach has been proven as superior, so the management remains controversial. Arthroscopic surgery with subchondral drilling might be indicated in small lesions whereas bigger lesions >2 cm or multiple loose bodies should be approached with open surgery. Open surgery offers multiple possibilities such as refixation of the cartilage, autologous chondrocyte implantation or the use of a collagenous scaffold, usually in combination with removal of the underlying sclerotic bone [17, 18, 21, 26, 27].

Despite reported failures of OD refixation [28], this approach showed a better outcome in our case. The Tegner–Lysholm score as well as the MOCART showed a higher value for refixation (Lysholm 95, MOCART 75) in comparison to reconstruction and coverage with collagenous membrane (Lysholm 78, MOCART 65) at 1 year follow-up. These findings support the theory that the outcome after OD is dependent on the vascular situation and the cartilage surface. The more physiological the cartilage layer and the higher the stability, the better the outcome seems to be. Preserving the original cartilage layer should be the main goal although a collagenous membrane seems to produce promising results [29, 30]. Other prognostic outcome factors are lesion size, patient age and intensity of sclerosis [25, 31–33]. Although the literature offers several surgical options, there is no consensus on the best treatment.

The AMIC used in her right knee in this case is a safe and effective cartilage restoration technique. AMIC is a one-stage procedure combining microfracturing or subchondral drilling with the addition of a biological scaffold on top. Various authors have shown that patients undergoing cartilage repair with a collagenous matrix show a significant decrease in pain as well as improvements in different clinical scores. Follow-up MRI and re-arthroscopies documented satisfying results with integration of the scaffold [34].

Finally, this is the first case to present different surgical techniques in a single patient with bilateral OD of the medial femoral condyle. One year postoperative the knee with refixation of the OD lesions showed a significantly better result in comparison to the contralateral knee with subchondral drilling and collagenous membrane coverage in AMIC technique. A follow-up of 12 months may not display a final situation and further clinical change might occur over time.

## Conclusions

Non-operative treatment produces satisfactory results in young and active patients with OD lesions in their knee joints. A thorough investigation including diagnostics of the contralateral side due to possible bilateral OD should be taken into consideration. If conservative treatment fails, surgery should be taken into account in order to help juvenile patients to return to sports and regain quality of life. In case surgery is arranged, a preservation of the lesion, independent of the preferred operative technique, should be aspired. As we consider that reconstruction of the subchondral bone does not present an operative challenge, the focus in OD surgery should be preservation of the cartilaginous layer whenever possible or replacement with highest possible tissue quality, such as autologous chondrocyte implantation. In particular, the young benefit from such intervention with regard to sporting activities and social integration and in prevention of early onset osteoarthritis.

## Consent

Written informed consent was obtained from the patient for publication of this case report and any accompanying images. A copy of the written consent is available for review by the Editor-in-Chief of this journal.

## Abbreviations

AMIC: autologous matrix-induced chondrogenesis
MOCART: magnetic resonance observation of cartilage repair tissue
MRI: magnetic resonance imaging
OD: osteochondrosis dissecans
